# Function identification of miR159a, a positive regulator during poplar resistance to drought stress

**DOI:** 10.1093/hr/uhad221

**Published:** 2023-11-07

**Authors:** Tiantian Fu, Chun Wang, Yuzhang Yang, Xiaoqian Yang, Jing Wang, Lichun Zhang, Zeqi Wang, Yanwei Wang

**Affiliations:** State Key Laboratory of Tree Genetics and Breeding, National Engineering Research Center of Tree Breeding and Ecological Restoration, Key Laboratory of Genetics and Breeding in Forest Trees and Ornamental Plants, Ministry of Education, The Tree and Ornamental Plant Breeding and Biotechnology Laboratory of National Forestry and Grassland Administration, College of Biological Sciences and Biotechnology, Beijing Forestry University, Beijing 100083, China; State Key Laboratory of Tree Genetics and Breeding, National Engineering Research Center of Tree Breeding and Ecological Restoration, Key Laboratory of Genetics and Breeding in Forest Trees and Ornamental Plants, Ministry of Education, The Tree and Ornamental Plant Breeding and Biotechnology Laboratory of National Forestry and Grassland Administration, College of Biological Sciences and Biotechnology, Beijing Forestry University, Beijing 100083, China; State Key Laboratory of Tree Genetics and Breeding, National Engineering Research Center of Tree Breeding and Ecological Restoration, Key Laboratory of Genetics and Breeding in Forest Trees and Ornamental Plants, Ministry of Education, The Tree and Ornamental Plant Breeding and Biotechnology Laboratory of National Forestry and Grassland Administration, College of Biological Sciences and Biotechnology, Beijing Forestry University, Beijing 100083, China; State Key Laboratory of Tree Genetics and Breeding, National Engineering Research Center of Tree Breeding and Ecological Restoration, Key Laboratory of Genetics and Breeding in Forest Trees and Ornamental Plants, Ministry of Education, The Tree and Ornamental Plant Breeding and Biotechnology Laboratory of National Forestry and Grassland Administration, College of Biological Sciences and Biotechnology, Beijing Forestry University, Beijing 100083, China; State Key Laboratory of Tree Genetics and Breeding, National Engineering Research Center of Tree Breeding and Ecological Restoration, Key Laboratory of Genetics and Breeding in Forest Trees and Ornamental Plants, Ministry of Education, The Tree and Ornamental Plant Breeding and Biotechnology Laboratory of National Forestry and Grassland Administration, College of Biological Sciences and Biotechnology, Beijing Forestry University, Beijing 100083, China; State Key Laboratory of Tree Genetics and Breeding, National Engineering Research Center of Tree Breeding and Ecological Restoration, Key Laboratory of Genetics and Breeding in Forest Trees and Ornamental Plants, Ministry of Education, The Tree and Ornamental Plant Breeding and Biotechnology Laboratory of National Forestry and Grassland Administration, College of Biological Sciences and Biotechnology, Beijing Forestry University, Beijing 100083, China; State Key Laboratory of Tree Genetics and Breeding, National Engineering Research Center of Tree Breeding and Ecological Restoration, Key Laboratory of Genetics and Breeding in Forest Trees and Ornamental Plants, Ministry of Education, The Tree and Ornamental Plant Breeding and Biotechnology Laboratory of National Forestry and Grassland Administration, College of Biological Sciences and Biotechnology, Beijing Forestry University, Beijing 100083, China; State Key Laboratory of Tree Genetics and Breeding, National Engineering Research Center of Tree Breeding and Ecological Restoration, Key Laboratory of Genetics and Breeding in Forest Trees and Ornamental Plants, Ministry of Education, The Tree and Ornamental Plant Breeding and Biotechnology Laboratory of National Forestry and Grassland Administration, College of Biological Sciences and Biotechnology, Beijing Forestry University, Beijing 100083, China

## Abstract

Drought seriously affects the growth and development of plants. MiR159 is a highly conserved and abundant microRNA family that plays a crucial role in plant growth and stress responses. However, studies of its function in woody plants are still lacking. Here, the expression of miR159a was significantly upregulated after drought treatment in poplar, and the overexpression of miR159a (OX159a) significantly reduced the open area of the stomata and improved water-use efficiency in poplar. After drought treatment, OX159a lines had better scavenging ability of reactive oxygen species and damage of the membrane system was less than that in wild-type lines. *MYB* was the target gene of miR159a, as verified by psRNATarget prediction, RT–qPCR, degradome sequencing, and 5′ rapid amplification of cDNA ends (5′ RACE). Additionally, miR159a–short tandem target mimic suppression (STTM) poplar lines showed increased sensitivity to drought stress. Transcriptomic analysis comparing OX159a lines with wild-type lines revealed upregulation of a series of genes related to response to water deprivation and metabolite synthesis. Moreover, drought-responsive miR172d and miR398 were significantly upregulated and downregulated respectively in OX159a lines. This investigation demonstrated that miR159a played a key role in the tolerance of poplar to drought by reducing stomata open area, increasing the number and total area of xylem vessels, and enhancing water-use efficiency, and provided new insights into the role of plant miR159a and crucial candidate genes for the molecular breeding of trees with tolerance to drought stress.

## Introduction

Research on the resistance of plants to environmental stresses has received widespread attention [1]. Among the diverse stress stimulators, drought has an enormously destructive influence on plant growth and crop yields [2, 3]. With the increase in the number of drought areas, it is imperative to clarify the drought resistance mechanism of agricultural and forestry crops, and develop drought-tolerant crop varieties [4]. During long-term evolution, plants have developed diverse defensive mechanisms in responding to environmental stresses, especially microRNA (miRNA)-mediated regulation. miRNA is a small non-coding RNA with a length of 20–24 nucleotides, and is processed from endogenous miRNA precursors with a stem–loop structure [5]. It inhibits translation or cleaves target genes to negatively regulate post-transcriptional gene expression [6]. Reactive oxygen species (ROS), peroxidase (POD), mitogen-activated protein kinase (MAPK), and abscisic acid (ABA) are some of the significant signaling molecules mediated by several plant miRNAs [7]. These signaling molecules govern different plant adaptation processes. For example, miR472a can target the *NBS*-*LRR* gene to enhance poplar defense against *Cytospora chrysosperma* infection [8]. Besides, the expression levels of miR393, miR171, miR156, miR159, and miR164 were detected to undergo changes in response to mulberry powdery mildew infection in wheat cultivars [9]. Moreover, several studies proved the involvement of miRNAs in regulating abiotic stresses. For example, miR398 protected plants from oxidative damage by targeting Cu/Zn superoxide dismutases (*CSD1* and *CSD2*) during exposure to sulfur dioxide [10]. Plants with overexpression of miR393a exhibited high tolerance to drought stress [11], and miR169o could enhance drought resistance of *Populus* by targeting the *PtNF*-*YA6* gene [12]. MiR172d could enhance the drought tolerance of poplar by inhibiting the target gene *PuGTL1* and inducing *PuSDD1* expression to improve water-use efficiency (WUE) [13]. Some drought-responsive miRNA–mRNA modules were further confirmed to respond to drought stress. miR5200 was involved in regulating stomatal movement by inhibiting targets highly homologous to the flowering gene *FLOWERING LOCUS T* (FT) [14]. The miR824–AGL16 module similarly played a tremendous role in the satellite meristem of stomatal development [15]. However, knowledge of the regulatory mechanism of how miRNA regulates plant stomatal development and helps plants cope with drought stress is urgently needed for further elucidation. miR159 exhibits a typically conserved and abundant profile in plant species, and many studies have demonstrated that miR159 could respond to diverse plant stresses [16]. The regulation of expression of miR159 under abiotic stress, including drought stress, has been investigated in recent years. miRNA transcriptome analysis in bread wheat (*Triticum aestivum*) identified drought-responsive miRNAs including miR159 [17]. The sly–miR159–SlMYB33 module is associated with the accumulation of two compounds, proline and putrescine, in tomato (*Solanum lycopersicum*) under drought stress [18]. Previous studies confirmed that miR159 also accumulated to higher levels in maize, wheat, and barley under drought stress [19]. Such studies on miR159 in diverse plant species suggested that miR159 might play crucial roles in drought responses. However, the functional validation and investigation of miR159 regulation in drought stress have been poorly studied in plants, including trees, so far.

Poplar is widely used as a model tree species for tree genetic improvement due to its fast growth rate, high yield, and efficient regeneration [20]. It has a great need for water because of its high growth capability, but often suffers from drought stress on account of water shortage in most areas, which seriously affects its survival. Consequently, it is imperative to identify drought response genes based on poplar characteristics and clarify the regulation of drought resistance in poplar. The mechanisms underlying drought tolerance in poplar have been extensively studied recently, including the molecular functions of key regulatory factors such as transcription factors and miRNAs [11–13, 21–23]. Previous investigations found that miR159 was highly expressed in maize, wheat, barley, and cabbage under drought stress [24]. However, there are few studies on the response of miR159 to abiotic stress in poplar, and its regulatory mechanism remains unclear. In this study, multifunctional miR159a was analyzed in poplar using transgenic technology. We discovered that miR159a overexpression reduced the open stomatal area of transgenic plants and increased the number and total area of xylem vessels, which enhanced drought tolerance and positively controlled WUE levels. Additionally, transcriptome data of OX159a transgenic lines and wild type (WT) were then analyzed to investigate miRNAs and their target genes involved in drought tolerance. Intriguingly, our investigation showed the combined effects of miR159a overexpression on other miRNAs, its target genes, and some functional genes related to drought resistance and development of poplar. Overall, our investigation experimentally revealed the function of miR159a in drought tolerance and further clarified the response mechanism in poplar, providing insights into the theoretical and empirical bases and genetic resources for molecular breeding of trees with resistance to drought stress.

## Results

### Identification and expression analysis of miR159a

**Figure 1:**
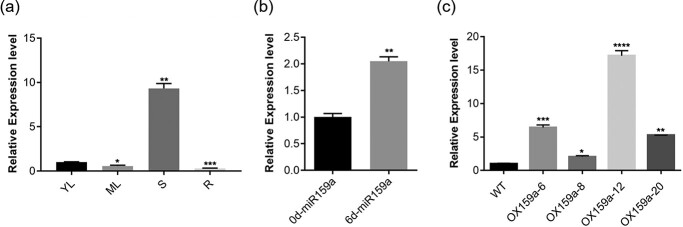
Identification and expression analysis of miR159a. **a** Relative expression of miR159a in young leaves (YL), mature leaves (ML), stems (S), and roots (R) was determined by RT–qPCR. The expression of YL was set to 1. **b** Changes in miR159a expression after drought treatment. The value for the untreated group (0 day) was set as 1. **c** Identification of miR159a transgenic poplar. The expression level of transgenic lines was determined using RT–qPCR. The internal control was established with 5.8S rRNA. Error bars: ± standard deviation with three biological replicates. *P < .05; **P < .01; ***P < .001; ****P < .0001 (Student’s *t*-test).

The expression of miR159a needed to be determined among different tissues of poplar. Hence, we selected 45-day WT tissue culture poplar plants, extracted small RNA from young leaves, mature leaves, stems, and roots and then performed reverse transcription–quantitative polymerase chain reaction (RT–qPCR) experiments on miR159a. We found that miR159a had the highest expression level in stems and the lowest expression level in roots (Fig. 1a). Compared with young leaves, the expression of miR159a in stems was upregulated by 9.33 times and downregulated in roots by 2.57 times. The differential expression of miR159a was not significant (decreased 0.75 times) in mature leaves compared with young leaves. We performed miR159a RT–qPCR using WT lines under normal and drought treatment conditions to further investigate whether miR159a responded to drought stress. It was found that miR159a showed an accumulation trend after drought stress compared with normal conditions, indicating that miR159a had a positive feedback effect under drought stress (Fig. 1b).

**Figure 2:**
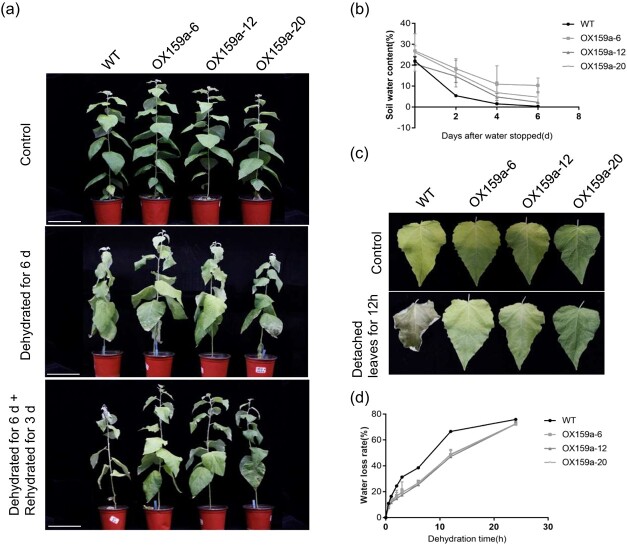
MiR159a overexpression increased poplar drought tolerance. **a** Phenotypes of WT and OX159a lines 0 and 6 days after stopping watering and after rehydration for 3 days. Scale bar = 12 cm. **b** SWC in OX159a and WT lines under drought stress. **c**, **d** Water loss rate in leaves of OX159a and WT lines in the *ex vivo* state. Error bars: ± standard deviation with three biological replicates.

Then, we performed genetic transformation of poplar using miR159a precursor and obtained four transgenic poplar lines with miR159a overexpression (OX159a), OX159a-6, OX159a-8, OX159a-12, and OX159a-20, via *Agrobacterium*-mediated transformation of poplar 84K (*Populus alba × Populus glandulosa*) leaves to further examine the regulatory function of poplar miR159a under drought stress. Among these transgenic poplar lines, the expression level in OX159a-12 lines was significantly higher (17-fold) than that in WT lines. Besides, the increased expression level of the other two lines was 6- and 5-fold, whereas the expression level of OX159a-8 lines was not high (2-fold) and excluded for the following analysis (Fig. 1c).

### Overexpression of miR159a enhanced tolerance to drought stress

Transgenic miR159a lines (OX159a-6, OX159a-12, and OX159a-20) and WT were selected for further drought treatment to study the function of miR159a in poplar under drought stress. We compared the phenotypic changes, including leaf wilting degree, wilting duration, and soil water content (SWC), of WT and OX159a lines before drought treatment (0 days), dehydrated for 6 days, and rehydrated for 3 days. All WT leaves had wilted on the sixth day after watering was stopped; however, no obvious change was observed in transgenic lines. Furthermore, the OX159a plants recovered rapidly and returned to normal growth after 3 days of rehydration; however, the shoot apices of WT lines did not recover (Fig. 2a).

Then, we continuously measured the SWC of each pot of treated plants at 0, 2, 4, and 6 days after stopping watering. The SWC decreased, indicating that both WT and OX159a lines underwent rapid water loss after drought treatment. Intriguingly, during the drought stress treatment we found that the SWC of OX159a lines was higher than that of WT lines, and the latter had a higher sensitivity to drought (Fig. 2b), suggesting that the miR159a-overexpression lines were more drought-tolerant than WT.

For further validation, fresh detached leaves were weighed for water loss assay to investigate the physiological response of the drought-tolerance phenotype of miR159a in leaves. The results showed that the water loss rate of OX159a lines was obviously lower than that of WT lines after the plant leaves were detached. WT fresh weight decreased by 66.63 ± 0.26%, but the fresh weight of three OX159a lines reduced by only 48.51 ± 2.3%, 47.07 ± 0.97%, and 49.02 ± 2.65% after 12 h of treatment (Fig. 2c and d). This result suggested that the enhanced water retention ability of the OX159a lines was one of the reasons for the emergence of the drought-tolerant phenotype.

### Involvement of miR159a overexpression in regulating ROS accumulation under drought conditions

**Figure 3:**
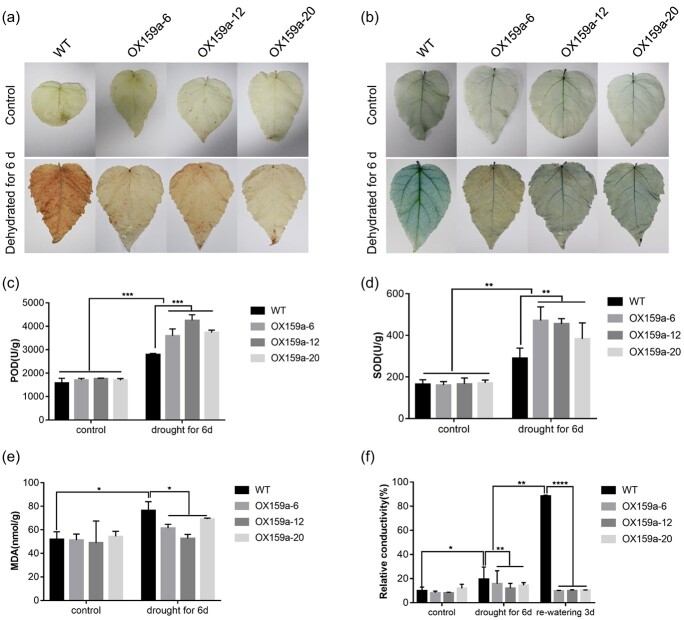
MiR159a overexpression enhanced ROS scavenging ability and decreased the degree of membrane damage. **a** DAB staining for histochemical detection of hydrogen peroxide. **b** Histochemical superoxide detection using trypan blue staining. **c** POD activity. **d** SOD activity. **e** MDA content. **f** REC in OX159a and WT lines under drought stress. Error bars: ± standard deviation with three biological replicates. *P < .05; **P < .01; ***P < .001; ****P < .0001 [ANOVA with *post hoc* Bonferroni correction (*P* < .05)].

Drought stress accelerates the accumulation of ROS, thereby destroying the plant’s homeostasis. However, efficient ROS scavenging ability improves the drought resistance of plants. We performed 3,3′-diaminobenzidine (DAB) staining on leaves of OX159a and WT lines before and after drought treatment to investigate whether the overexpression of miR159a could influence the degradation of superfluous hydrogen peroxide (H_2_O_2_) and superoxide in poplar. Histochemical analyses indicated no remarkable difference between OX159a and WT lines before drought treatment. However, the WT line had a darker brown color than the OX159a line once subjected to drought stress, indicating that WT plants accumulated more H_2_O_2_ after drought, while H_2_O_2_ accumulation was compromised in OX159a lines (Fig. 3a). We also performed trypan blue staining on the leaves of poplar under normal and drought stress to observe the living status of the leaves. Through the trypan blue staining experiment, it was found that the WT plants had more dead cells than the OX159a plants after drought treatment (Fig. 3b). Thus, trypan blue staining further verified that OX159a lines demonstrated enhanced resistance under drought conditions.

Then, we measured the activity of antioxidant enzymes for validation. Superoxide dismutase (SOD) and POD are essential antioxidants for scavenging ROS. The activities of these two enzymes were investigated using OX159a and WT lines before and after drought treatment. SOD and POD activities were not significantly different between the OX159a and WT lines under adequate water conditions. After 6 days of drought treatment, the levels of both enzymes increased in OX159a and WT lines, but POD and SOD contents were significantly higher in OX159a lines than in WT (Fig. 3c and d). Overall, the overexpression of miR159a in poplar caused better tolerance to drought stress.

We further measured the relative conductivity (REC) and malondialdehyde (MDA) content to investigate whether the difference in the degree of cell membrane damage in OX159a lines and WT was influenced by drought stress. The MDA content increased to 76.5 nmol/g (WT), 61.5 nmol/g (OX159a-6), 52.7 nmol/g (OX159a-12), and 69.1 nmol/g (OX159a-20) after drought treatment, suggesting that the WT was more damaged than OX159a lines (Fig. 3e). After 6 days of dehydration, the REC of OX159a lines was significantly lower than that of WT lines. On day 3 of rehydration, the REC of WT lines was still increased and was significantly higher than that of OX159a lines. The electrical conductivity of OX159a lines gradually recovered to the state with no stress (Fig. 3f). Overall, the cell membrane of WT lines was more seriously damaged than that of OX159a lines under water shortage.

### Overexpression of miR159a reduced transpiration and photosynthetic activity of poplar

**Figure 4:**
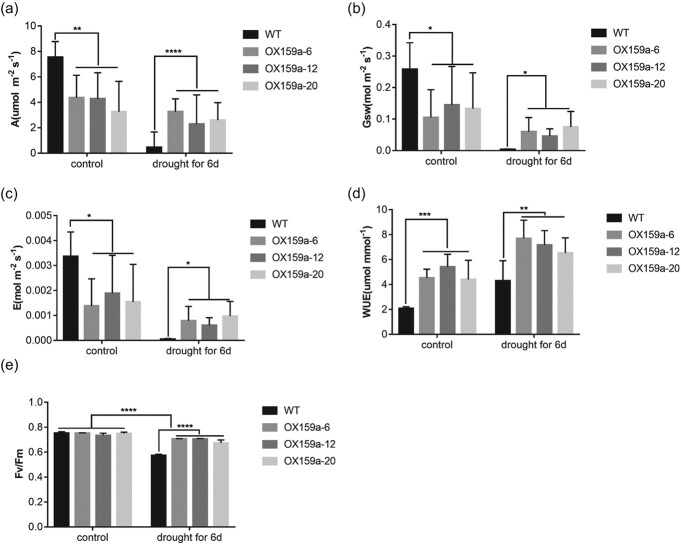
MiR159a overexpression reduced transpiration and photosynthetic activity in poplar. **a** Net photosynthetic rate (*A*). **b** Stomatal conductance (Gsw). **c** Leaf transpiration rate (*E*). **d** Instantaneous WUE. **e***F*_v_*/F*_m_ of OX159a and WT plants at 400 μM/mol CO_2_ concentration under normal conditions (0 days) and drought treatment (6 days). Error bars: ± standard deviation with three biological replicates. *P < .05; **P < .01; ***P < .001; ****P < .0001 [ANOVA and *post hoc* Bonferroni correction (*P* < .05)].

Gas-exchange parameters were examined in 45-day-old OX159a and WT lines to further investigate the effect of miR159a overexpression on the photosynthetic activity of poplars. The net photosynthetic rate (*A*) indicated that the OX159a lines had a lower photosynthetic capacity than the WT lines (Fig. 4a). The stomatal conductance (Gsw) of the OX159a lines was lower than that of the WT lines under the same CO_2_ conditions (Fig. 4b), and the leaf transpiration rate (*E*) in these plants was also lower. Various photosynthetic indexes were measured during drought stress treatment to better clarify the difference between transgenic poplar and WT. Under drought treatment, the gas-exchange parameters differed significantly between OX159a and WT plants in responding to drought stress. *A* decreased more rapidly in WT lines than in OX159a lines (Fig. 4a). Gsw (Fig. 4b) and *E* (Fig. 4c) showed similar patterns, with a smaller decline in OX159a lines compared with WT lines. Interestingly, the instantaneous WUE (*A/E*) values of OX159a lines were higher than those of WT lines (Fig. 4d).

*F*_v_ */F*_m_ reflects the efficiency of photosystem II (PS II) in converting absorbed light energy into chemical energy, which is the primary light energy conversion efficiency [25, 26]. Drought stress can lead to a decrease in both net photosynthetic rate and *F*_v_*/F*_m_. In our study, the differences in *F*_v_*/F_m_* between lines were not statistically significant and were all slightly below 0.8 under normal growth conditions. Under 6 days of drought stress, *F*_v_*/F*_m_ was significantly higher in the OX159a lines than in WT lines (Fig. 4e).

### MiR159a overexpression reduced open area of stomata and enhanced vessel development

**Figure 5:**
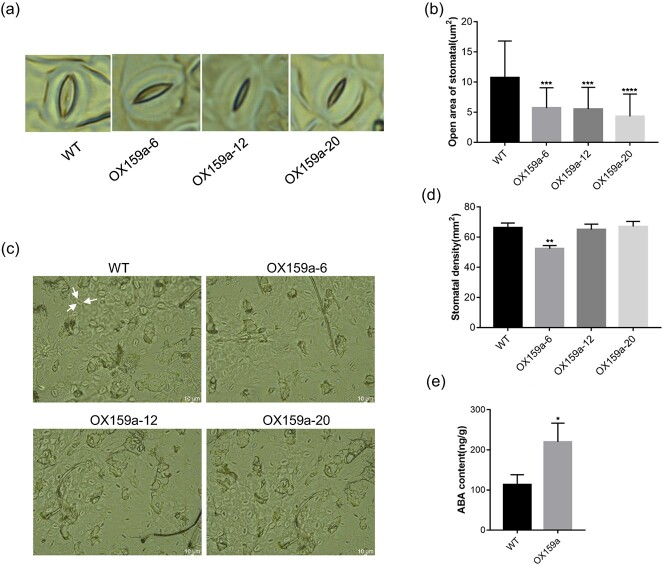
Representative images of abaxial epidermis of mature leaves of WT and OX159a lines. **a**, **b** Stomatal open area. **c**, **d** Stomatal density. **e** ABA content of WT and OX159a plants under normal conditions. The OX159a-12 line was used taking into account the highest expression of miR159a. Three biological replicates, *n* = 10 leaves, error bars: ± standard deviation (based on measurements in three different regions of leaves). *P < .05; **P < .01; ***P < .001; ****P < .0001 (Student’s *t*-test).

Stomata of OX159a and WT lines were observed using a microscope to examine whether miR159a affected stomata parameters. Interestingly, OX159a lines showed a decreased open area of stomata in the leaf abaxial epidermis (Fig. 5a and b). However, the stomatal density of the transgenic lines was not significantly different from that of the WT lines except for the OX159a-6 line. (Fig. 5c and d). These results demonstrated that miR159a negatively controlled the open area of stomata in poplar. The ABA contents were then determined in OX159a and WT lines to further investigate the reasons for the difference in stomatal opening. Under normal conditions, the OX159a lines had significantly higher ABA content than the WT lines, indicating that miR159a overexpression increased ABA accumulation (Fig. 5e).

Tissue sections of OX159a and WT stems were sampled and chemically stained considering that miR159a exhibited the highest expression level in poplar stem. Then, the sections were scanned using a scanning electron microscope to visualize the difference in vessel cells between the OX159a and WT lines (Supplementary Data Fig. S1a). Intriguingly, the total areas of vessels in the OX159a lines were larger than in WT lines (Supplementary Data Fig. S1b and c), and the number of vessels in the OX159a lines was significantly higher than in the WT line (Supplementary Data Fig. S1d and e). The increases in areas and number of vessels might contribute to more effective water transport in plants. The upregulation of the gene *NAC007* [27] (*Pop_A07G006579*), which is involved in vessel development, in OX159a supported this finding. Overall, all these phenotypes of OX159a lines typically associated with drought tolerance supported the positive function of miR159a in drought regulation.

### Transcriptomic revealed differentially expressed genes and phenotypical traits in OX159a lines compared with WT lines

**Figure 6:**
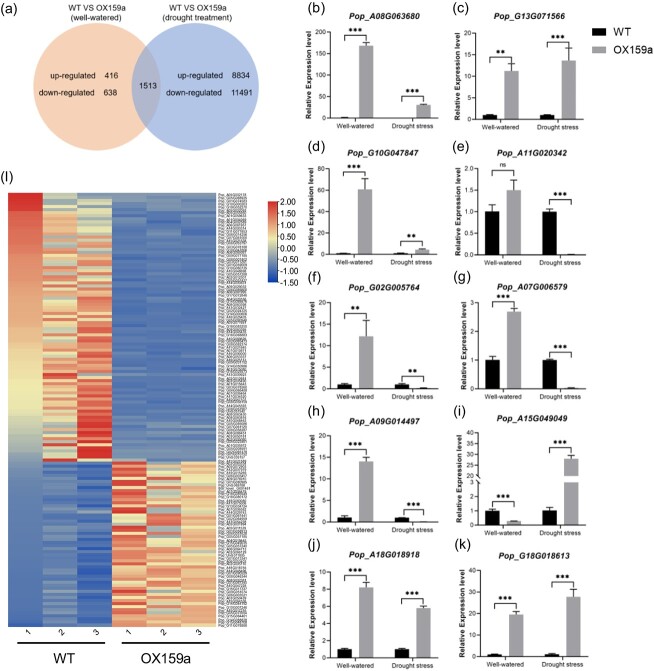
DEGs in OX159a and WT plants under well-watered and drought conditions. Transcriptome analysis was performed on mature leaves (fifth and sixth leaves) of 45-day-old plants well-watered or subjected to drought for 6 days. **a** Venn diagram of DEGs between treatments. **b**–**k** Verification of DEGs as determined by RT–qPCR. 18S rRNA was used as the internal control. **l** Heat map of DEGs associated with drought stress (|log2| ≥ 2). The OX159a-12 line was used for the analysis taking into account the highest expression of miR159a. Error bars: ± standard deviation with three biological replicates. **P < .01; ***P < .001; ns, not significant (Student’s *t*-test).

We performed transcriptomic analysis using 45-day-old OX159a and WT lines, which were grown under well-watered conditions or subjected to 6 days of drought stress. Under well-watered conditions, 2567 genes showed differential expression levels (*P* < .05) (Fig. 6a, Supplementary Data Table S1). We identified 72 differentially expressed genes (DEGs) related to abiotic stress in OX159a lines, including 18 DEGs related to drought (Supplementary Data Table S2). Gene Ontology (GO) analysis revealed that terms at the biological process level were mainly associated with ‘photosynthesis’ (GO:0015979), ‘negative regulation of gene expression, epigenetic’ (GO:0045814), ‘response to light stimulus’ (GO:0009416), ‘response to abiotic stimulus’ (GO:0009628), ‘response to osmotic stress’ (GO:0006970), and ‘negative regulation of cellular process’ (GO:0048523) (Supplementary Data Table S3).

The expression of a large number of DEGs significantly increased after the drought treatment, with 21 838 genes differentially expressed in the OX159a lines compared with the WT lines (Fig. 6a, Supplementary Data Table S4). To validate the accuracy of the RNA-seq, we used the RT–qPCR method to analyze 10 upregulated and downregulated DEGs related to photosynthesis and drought under well-watered conditions and 6 days of drought treatment. The results showed that the expression patterns of all the genes highly matched the differential fold changes determined by RNA-seq, indicating the reliability of DEG identification based on RNA-seq data (Fig. 6b–k). GO terms of the DEGs at the biological process level were primarily related to ‘photosynthesis’ (GO:0015979), most of which were related to ‘photosynthesis, light-harvesting in photosystem I’ (GO:0009768) and ‘photosynthesis, light harvesting’ (GO:0009765). The expression of an array of genes annotated to ‘regulation of DNA-templated transcription in response to stress’ (GO:0043620), ‘response to abiotic stimulus’ (GO:0009628), and ‘response to water deprivation’ (GO:0009414) were induced in the OX159a plants (Supplementary Data Table S5). A clustering heat map for DEGs associated with drought stress and post-drought stress was then constructed (Fig. 6l, Supplementary Data Table S6). Interestingly, we found that a range of well-known genes related to abiotic stress were upregulated in the OX159a lines under drought conditions, including *MYB27*, *bHLH122*, *GAI* (DELLA protein), *BBX24*, *BPM* (BTB/POZ-MATH), *CAB*, and *HY5* (Supplementary Data Table S7) [28–32].

Furthermore, we analyzed the Kyoto Encyclopedia of Genes and Genomes (KEGG) pathway of these DEGs and found that the most important enriched pathways involved carbon metabolism (ko01200), photosynthesis (ko00195), starch and sucrose metabolism (ko00500), porphyrin and chlorophyll metabolism (ko00860), carbon fixation in photosynthetic organisms (ko00710), photosynthesis-antenna proteins (ko00196), glyoxylate and dicarboxylate metabolism (ko00630), and so forth (Supplementary Data Fig. S2). Interestingly, we found that the MAPK signaling pathway involved in abiotic stress was enriched in the OX159a lines after drought stress (Supplementary Data Fig. S3).

### Target gene analysis of miR159a in poplar

**Figure 7:**
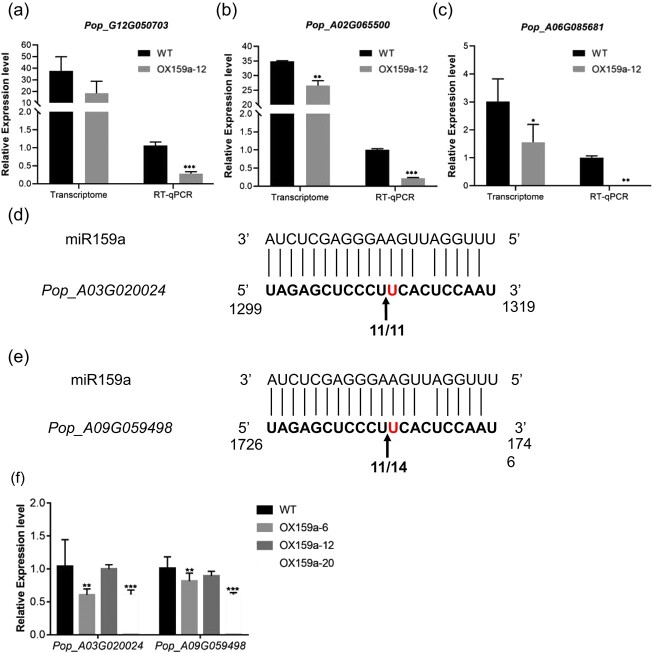
Detection and validation of miR159a in poplar. **a** Expression level of *Pop_G12G050703* (LHY) between OX159a-12 and WT lines. The OX159a-12 line was used for the analysis, considering the highest expression of miR159a. **b** Expression level of *Pop_A02G065500* (TNNI3K) in OX159a-12 and WT lines. **c** Expression level of *Pop_A06G085681* in OX159a-12 and WT lines. **d**, **e** Target complementary sequences and cleavage sites of miR159a. The cleavage sites, which are located between the 10th and 11th nucleotides of the miRNA, are denoted by arrows. **f** Expression abundance of the target gene in OX159a lines. Expression levels of genes were determined by RT–qPCR. 18S rRNA was used as the internal reference gene. Error bars: ± standard deviation with three biological replicates. *P < .05; **P < .01; ***P < .001 (Student’s *t*-test).

MiR159 has been extensively found to target the *MYB* gene family in other plants, and plays important roles in plant growth and development, hormone signaling, light signal transduction, and response to environmental stresses [33–41]. The target genes were predicted to further investigate the molecular mechanism of miR159a to validate and determine which candidate *MYB* genes were targeted by miR159a in poplar. As a result, 196 target genes were predicted by psRNATarget (Supplementary Data Table S8), including *Pop_A03G020024* and *Pop_A09G059498*, which were annotated to transcription factor MYB, *Pop_G12G050703* annotated to MYB-related transcription factor LHY, *Pop_A02G065500* annotated to serine/threonine-protein kinase TNNI3K, and *Pop_A06G085681* annotated to POD. Considering that miR159a was the most highly expressed in the OX159a-12 lines, we selected WT and OX159a-12 lines for RT–qPCR analysis of the expression levels of these targets. Their expression levels of these targets in OX159a-12 lines were significantly lower than those of WT lines (Fig. 7a–c and f). Additionally, through transcriptomic analysis (RNA-seq) we also found the predicted target genes were downregulated in WT lines under drought treatment (Table 1). Similar expression patterns were also found in OX159a transgenic plants (Table 1). These findings suggested that these genes were potential targets of miR159a.

**Table 1:** Transcriptome analysis of target genes related to drought stress in OX159a and WT lines.

Gene ID	0d-OX159a-12 FPKM	0d-WT FPKM	6d-WT FPKM	Functional annotation	Homologs of *Arabidopsis thaliana*
*Pop_G12G050703*	18.39	37.60	2.72	MYB-related transcription factor	*AT1G18330*
*Pop_A02G065500*	26.60	34.89	18.68	Serine/threonine-protein kinase	*AT3G22750*
*Pop_A06G085681*	1.56	3.01	2.6	Peroxidase	*AT2G37130*
*Pop_A09G059498*	0.84	1.15	0.85	Transcription factor MYB	*AT3G11440*
*Pop_A11G021726*	142.28	204.07	92.46	Methionine sulfoxide reductase	*AT1G53670*

Then, the analysis of poplar degradome sequencing indicated that eight genes were detected as the cleavage sites of miR159a (Table 2). Interestingly, we found that *Pop_A03G020024* and *Pop_A09G059498* were both confirmed by psRNATarget prediction and degradome sequencing. The two genes were further validated to be targeted by miR159a via 5′ rapid amplification of cDNA ends (5′ RACE). The binding sites of miR159a located at the 3′ UTRs of the aforementioned two genes and 5′ RACE products were obtained through RT–PCR, which exactly matched the sequence of the *Pop_A03G020024* and *Pop_A09G059498* 3′ UTR. The two genes were cleaved at the 10th and 11th bases, corresponding to the 5′ end of the mature miR159a sequence, which was the typical cleavage site of miRNA on plant target genes (Fig. 7d and e).

**Table 2:** Targets of miR159 verified by degradome sequencing in poplar 84K.

Target transcript	T start	T stop	T slice	Allen score	Target annotation	Homologs of *Arabidopsis thaliana*
*Pop_A01G015461*	656	676	667	3.5	Unannotation	*AT4G27330*
*Pop_A01G054486*	1187	1207	1198	3	Transcription factor GAMYB-like	*AT5G06100*
*Pop_A03G020024*	1299	1319	1310	3	Transcription factor MYB	*AT5G06100*
*Pop_A08G063902*	1795	1816	1806	5	ACT domain-containing protein	*AT1G69040*
*Pop_A09G059498*	1726	1746	1737	3	Transcription factor GAMYB	*AT3G11440*
*Pop_G01G059116*	1718	1738	1729	3	Transcription factor GAMYB-like	*AT1G21840*
*Pop_G02G065007*	1599	1618	1609	4.5	Tetratricopeptide repeat protein SKI3	*AT1G76630*
*Pop_UnG026796*	1370	1389	1380	4.5	Tetratricopeptide repeat protein SKI3	*AT1G76630*

RT–qPCR was then performed using target genes to identify the changes in their expression levels to further verify the cleavage activity of miR159a in transgenic plants, including *Pop_A03G020024* and *Pop_A09G059498*. RT–qPCR using WT and OX159a lines showed that the expression level of the two target genes was significantly reduced (Fig. 7f), which was consistent with the expected negative regulation mode of miR159a and further supported *Pop_A03G020024* and *Pop_A09G059498* encoding *MYB* as targets.

### MiR159a suppression conferred reduced drought tolerance of poplar

**Figure 8:**
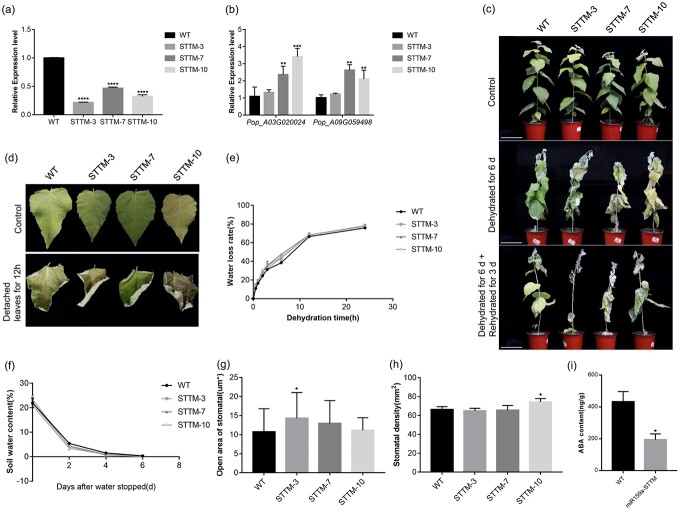
MiR159a-STTM decreased drought tolerance in poplar. **a** Identification of miR159a-STTM transgenic poplar. The expression level of miR159a-STTM lines was determined by RT–qPCR. 5.8S rRNA was used as the internal control. **b** Target gene expression in miR159a-STTM lines. 18S rRNA was used as the internal control. **c** Phenotypes of WT and miR159a-STTM lines 0 and 6 days after stopping watering and rehydration for 3 days under drought stress. Scale bar = 12 cm. **d**, **e** Dehydration speed of detached leaves from miR159a-STTM and WT lines under drought stress. **f** SWC in OX159a and WT lines under drought conditions. **g** Stomatal open area. **h** Stomatal density. *n* = 10 leaves (three different regions of each leaf were measured). **i** ABA content of WT and miR159a-STTM-3 plants under normal conditions. Error bars: ± standard deviation with three biological replicates. *P < .05; **P < .01; ***P < .001; ****P < .0001 (Student’s *t*-test).

To further validate the function of miR159a under drought stress, we generated miR159a-STTM transgenic lines, including STTM-3, STTM-7, and STTM-10, with suppression of miR159a using short tandem target mimic (STTM) technology (Fig. 8a). The expression level of the *Pop_A03G020024* and *Pop_A09G059498* transcripts were significantly higher in miR159a-STTM lines than in the WT lines (Fig. 8b). After drought stress, the leaves of miR159a-STTM lines appeared to be more wilted compared with those of WT lines (Fig. 8c). Furthermore, we measured the water loss rate of detached leaves and SWC differences between STTM and WT lines under drought treatment. The results showed that the detached leaves of STTM lines had a higher water loss rate (Fig. 8d and e), and the SWC in STTM lines was lower than that in WT lines (Fig. 8f). Additionally, stomatal measurement revealed that the stomatal aperture area increased by 3.6–33.1% in the miR159a-STTM lines compared with WT lines (Fig. 8g). However, there was no significant difference in stomatal density between miR159a-STTM and WT lines (Fig. 8h). ABA hormone content was also measured in both WT and miR159a-STTM lines. Under normal conditions, miR159a-STTM lines exhibited a significantly lower ABA content compared with WT lines (Fig. 8i). To investigate the relationship between miR159a and vessel cells, stem sections of miR159a-STTM and WT plants were sampled and stained. Scanning electron microscopy was used to visualize the vessels in the transgenic and WT lines. The total vessel area in miR159a-STTM lines was slightly smaller than that in WT lines, and similarly the number of vessels in miR159a-STTM lines was lower, although these differences were not significant (Supplementary Data Fig. S1c and d).

### Overexpression of miR159a altered expression levels of other miRNAs affecting the traits of poplars

**Figure 9:**
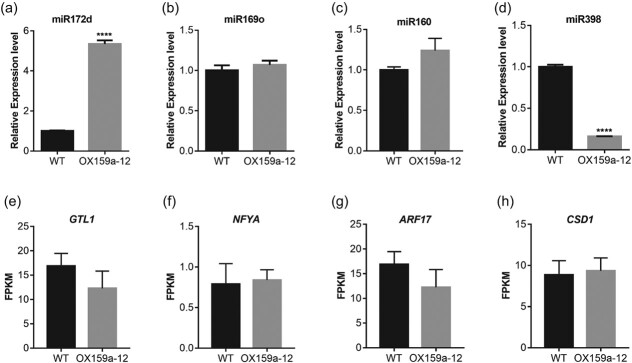
MiR159a affected the expression levels of drought-responsive miRNAs in poplar: (**a**) miR172d; (**b**) miR169o; (**c**) miR160; (**d**) miR398; (**e**) *GTL1*; (**f**) *NFYA*; (**g**) *ARF17*; and (**h**) *CSD1*. The OX159a-12 line was used for the analysis taking into account the highest expression of miR159a. Error bars: ± standard deviation with three biological replicates. The asterisk symbol represents significant differences ****P < .0001 (Student’s *t*-test).

Previous studies have suggested that miR172d, miR169o, miR160, and miR398 have the potential to participate in drought tolerance [12, 13, 42, 43]. In this investigation, miRNA was further extracted from 45-day-old OX159a and WT leaves and reverse-transcribed into cDNA for RT–qPCR to explore the role of these miRNAs in the drought resistance of the transgenic lines. The results showed that the expression level of miR172d increased dramatically, but not significantly for miR169o and miR160 in OX159a lines (Fig. 9a–c). In contrast, the expression level of miR398 decreased dramatically in transgenic lines (Fig. 9d). Then, we analyzed the changes in the expression of these miRNA targets in transcriptome data and found that the target gene *GTL1* (miR172d), and target gene *ARF17* (miR160) showed a downregulated pattern, although not a significant one (Fig. 9e–g). In contrast, the expression of the target gene *CSD1* (miR398b) was slightly upregulated (Fig. 9h).

## Discussion

Poplar provides raw materials for wood and paper industries globally, but poplar growth has been restricted by drought in arid regions [44]. Therefore, it is necessary to understand the molecular regulatory mechanisms of poplar response to drought. miRNAs and their target genes play a central role in responses to various biotic or abiotic stimuli. Among these, miR159a is an ancient multipurpose miRNA that mediates plant responses to different abiotic stresses [19, 45]. However, the molecular mechanisms of miR159a and its target genes regulating abiotic stress, especially drought response, in woody plants remain poorly understood.

In the present study, miR159a, as a positive regulator, enhanced the tolerance of poplar to drought stress. This provided a further clue for understanding the role of miR159a in coordinating poplar drought response and a novel reference for further research on miRNAs in poplar.

### Overexpression of miR159a improved drought resistance in transgenic poplars

MiR159a is evolutionarily conserved and has been reported to be responsive to drought stress in wheat, barley, and cabbage in previous studies [16], suggesting that increased expression levels of miR159 may enhance drought stress tolerance of plants. In contrast, miR159 levels decrease in several species under drought stress conditions [19, 46]. Cap-binding 80 protein expression was downregulated, miR159 levels decreased, and GAMYB-like homolog mRNA levels increased in potatoes [47]. These studies suggest that the response of this pathway to drought stress varies among plant species. In this study, we successfully obtained transgenic poplar plants that overexpress miR159a or inhibit the expression of miR159a. This verified the positive role of miR159a in drought tolerance of poplar. Drought significantly increased the expression of miR159a. Overexpression of miR159a significantly reduced stomatal aperture, improving WUE and drought resistance of poplar. In contrast, miR159a-STTM lines exhibited opposite phenotypes to OX159a lines (Figs 5b and 8g and h). OPEN STOMATA1 (*OST1*), a phytohormone-activated SNF-related protein kinase, and the OST1 domain can integrate ABA and osmotic stress signals through interacting with the *PP2C* family [48–52]. They are key mediators of stomatal closure and coping with hypertonic stress [50]. *OST1* and *PP2CA* expression was significantly higher in OX159a lines than in WT lines under normal growth conditions (Supplementary Data Fig. S4). Hence, we propose that miR159a could affect stomatal closure factors to reduce the stomatal open area for drought defense (Fig. 5b).

The structure and size of xylem vessels, which serve as conducting cells, are crucial factors affecting water transport in plants and determining their drought tolerance [27, 53]. Our tissue-specific experiment revealed that miR159a exhibited the highest expression in the stem. In order to investigate whether changes in miR159a expression would impact the morphology and characteristics of vessels in poplar stems, and then potentially affect their drought resistance, we utilized chemical staining to observe vessel number and morphology in 3-month-old poplar stem tissue sections. The results indicated that the total vessel areas in the OX159a lines were larger compared with the WT lines, and the number of vessels in the OX159a lines was significantly higher as well. Conversely, the total vessel area in miR159a-STTM lines was slightly smaller than in the WT lines, although not significantly so (Supplementary Data Fig. S1c). Similarly, the number of vessels in miR159a-STTM lines was slightly lower than in the WT lines (Supplementary Data Fig. S1d). The increased vessel areas and numbers may contribute to more efficient water transport in plants. These findings are consistent with the observation of a more drought-tolerant phenotype in the OX159a lines compared with the WT lines. A crucial piece of evidence explaining this cytological phenotype change is that the overexpression of miR159a led to an increased transcription level of *NAC007* (*Pop_A07G006579*) [27] (Fig. 6g), which is involved in vessel development, whereas the transcription level of *NAC007* did not show a significant decrease in miR159a-STTM plants (Supplementary Data Fig. S1f).

Excessive accumulation of ROS causes severe peroxidative damage (oxidative stress) to plant cell membranes and biological systems [54, 55]. Reactive oxygen removal efficiency is a crucial indicator of plant drought resistance [56, 57]. In our study, DAB staining indicated that WT lines under drought stress accumulated large amounts of ROS, Further, assays revealed a downregulation of POD and SOD enzyme activities (Fig. 3a, c and d). Additionally, the REC and MDA contents were low in OX159a lines compared with WT lines under drought stress (Fig. 3e and f). Moreover, the transcriptome data revealed differential expression of the *LHY* gene between OX159a and WT lines. *LHY* homologs have been confirmed to be related to drought tolerance in soybean (Table 1) [58]. In a previous study, the methionine sulfoxide reductase (*MSR*) gene was identified as having a cleavage site for miR159a and to play a role in ROS accumulation [59]. Hence, we examined the *MSR* gene in our transcriptome data and observed that its expression was significantly reduced in the OX159a lines (Table 1). These results suggested that elevated miR159a levels caused the activation of other genes to detoxify excessive ROS, thus enhancing drought tolerance.

The maximal PSII quantum yield (*F*_v_*/F*_m_) indicated the plant’s maximum efficiency of converting light energy. Drought stress reduced the net photosynthesis rate and *F*_v_*/F*_m_, which was consistent with previous studies [25, 26]. The *F*_v_*/F*_m_ and photosynthetic efficiency of OX159a lines were higher than those of WT lines after 6 days of drought treatment (Fig. 4e). We used trypan blue staining to detect dead cells in the leaves after drought treatment to better reflect the state of the plant leaves. It was observed that the OX159a lines were stained lighter than the WT lines (Fig. 3b). The expression of a few downstream genes involved in ‘abiotic stimulus–response’ (GO:0009628) and ‘water deprivation response’ (GO:0009414) was upregulated with the overexpression of miR159a, providing significant evidence supporting the enhanced drought tolerance of the OX159a line (Supplementary Data Table S9). All these differences were caused by the differential expression of miR159a, indicating that miR159a indirectly decreased plant photosynthesis and water absorption to resist drought stress when the external water content was reduced.

### Genes encoding MYB transcription factors were targeted by miR159a

A previous study reported that miR159 directly targeted genes encoding MYB transcription factors [60]. Extensive previous studies on MYB transcription factors indicated their significance in abiotic stresses, such as salt, cold, and drought stresses [16]. In this study, we established that *MYB Pop_A03G020024* and *Pop_A09G059498* transcripts were cleaved by miR159a through 5′ RACE and degradome sequencing (Fig. 7d and e). Besides, the transcription levels of *Pop_A03G020024* and *Pop_A09G059498* demonstrated opposite expression compared with the expression of miR159a under stress (Fig. 7f). Hence, we concluded that miR159a targeted *Pop_A03G020024* and *Pop_A09G059498*. In addition, we performed expression analysis of other target genes detected by the transcriptome and degradome of poplar under drought stress (Tables 1 and2). We found that the expression levels of most targets decreased. *Pop_G12G050703* and *Pop_A02G065500* were significantly downregulated under drought stress (Supplementary Data Fig. S5a and b). The expression levels of *Pop_A06G085681*, *Pop_A11G021726*, *Pop_G02G065007*, and *Pop_UnG026796* decreased, although not significantly (Supplementary Data Fig. S5c–f). However, the expression levels of some targets, including *Pop_A08G063902*, *Pop_A01G054486*, and *Pop_G01G059116*, increased under drought stress (Supplementary Data Fig. S5g), though not significantly for the latter two genes (Supplementary Data Fig. S5g–i). However, their roles and regulation modes under drought stress and whether they have other molecular functions are unclear. It is necessary to conduct suppression and overexpression trials of the aforementioned genes to further ascertain their specific molecular regulation mechanisms under drought stress.

### Overexpression of miR159a altered expression levels of other microRNAs

miRNA–mRNA modules are at the center of gene regulation pathways in response to abiotic stresses, biotic stresses, and plant development because these mRNAs encode translation factors and F-box proteins [61]. A previous study showed that miRNAs played significant roles and multiple functions in responding to abiotic stress to ensure balance [62], and the expression of some miRNAs might change the expression of a few other miRNAs, thereby affecting the phenotype of plants [63].

In this study, the overexpression of miR159a affected the expression levels of other miRNAs involved in the drought response. Recent studies found that miR172d could enhance drought tolerance and improve WUE in poplar by suppressing the target gene *PuGTL1* and inducing the expression of *PuSDD1* [13]. It was found that the PtmiR169o–*PtNF*–*YA6* module regulated poplar growth and drought resistance [12]. Interestingly, higher levels of Mdm–miR160 improved drought resistance and the Mdm–miR160–*MdARF17*–*MdHYL1* mechanism positively regulated drought resistance in apple [43]. In our study, OX159a lines increased the expression of miR169o, miR160, and especially miR172d (Fig. 9a–c). Moreover, miR398b expression significantly decreased in OX159a lines (Fig. 9d). A previous study found that the expression of osa-miR397a/b, osa-miR398b, osa-miR408-5p, and osa-miR528-5p reduced in drought-tolerant varieties, but increased in drought-sensitive varieties [64]. We analyzed the expression of these miRNA target genes using transcriptomic data and found that the expression of *GTL1* and *ARF17* was downregulated in the transgenic lines (Fig. 9e–g).

miRNAs were extracted from 45-day-old OX159a, miR159a-STTM, and WT plants to further investigate the differences in miR172d, miR169o, miR398, and miR160 expression levels in miR159a transgenic lines. The results revealed that the expression levels of miR172d, miR160, and miR169o in the miR159a-STTM line were not significantly different from those in the WT line (Supplementary Data Fig. S6). Intriguingly, miR398 expression was significantly increased in the transgenic lines (Supplementary Data Fig. S6), which corresponded to the finding that miR398 expression significantly decreased in the OX159a line. Therefore, it was hypothesized that a regulatory relationship between miR159a and miR398 might exist; however, the specific interaction mechanism needs further investigation.

### Conclusions and future perspectives

**Figure 10:**
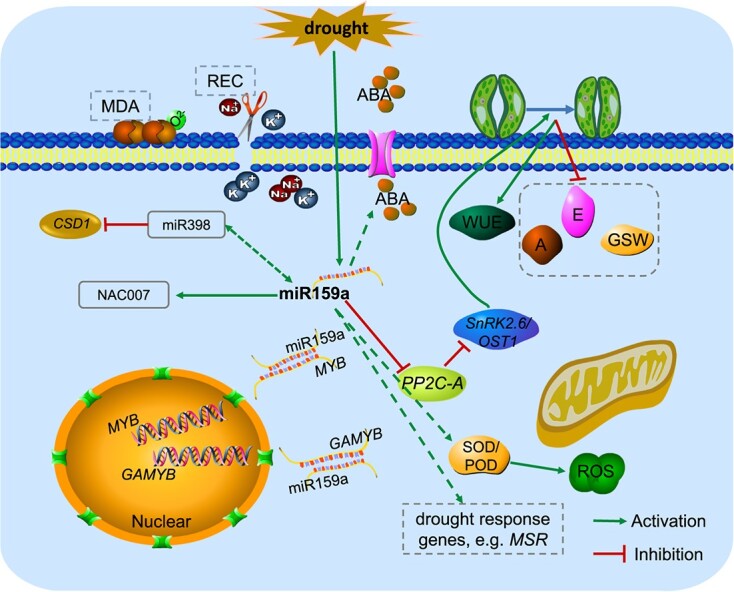
A model for enhancing drought resistance in poplar by miR159a overexpression. Overexpression of miR159a could reduce the open area of the stomata by increasing the expression level of the *OST1* gene. Overexpression of miR159a also could enhance the drought resistance of poplar by altering the expression level of miR398 and downstream genes related to the drought pathway. Increased expression of miR159a increased the cleavage of downstream *Pop_A03G020024* and *Pop_A09G059498* transcripts under drought stress, which enhanced ROS scavenging systems. *A*, net photosynthetic rate; *E*, transpiration rate of leaf; Gsw, stomatal conductance; REC, relative electrical conductivity; WUE, water use efficiency.

Drought stress is a major adversity for plant growth and seriously affects the sustainable development of forestry. Biotechnology or molecular marker-assisted breeding of new poplar varieties with drought tolerance or high water uptake efficiency is highly desirable. Previous studies have not found a consistent or unified role of miR159 in plant drought responses. These studies have mainly been limited to transcriptomic analysis, and functional validation experiments regarding the role of miR159a in drought have not been conducted. Therefore, further research is needed to explore the exact role and mechanisms of miR159 in plant drought responses. In this study, we observed that the miR159 family member miR159a was upregulated under drought treatment, and MYB transcription factors were identified as its target genes. The overexpression of miR159a in poplar increased the photosynthesis rate and decreased the water loss rate of leaves in enhancing resistance to drought stress, which was contrary to miR159a-STTM. Besides, miR159a overexpression also affected the expression level of other miRNAs in response to drought stress and downstream genes related to the drought pathway (Fig. 10). In summary, this study provided a potential strategy to improve plant WUE and drought resistance by regulating miRNA expression. Additionally, the interaction between miR159a and other miRNAs under drought stress may also be a research topic in the transcriptional regulation of downstream regulatory layers of miRNA159a. In woody plants, we only have a restricted comprehension of the epigenetic mechanisms, including miRNAs, that regulate drought resistance at transcriptional and post-transcriptional levels. More intensive research is needed to fill the gaps in our knowledge of these regulatory processes.

## Materials and methods

### Plant materials and growth conditions

84K (*P. alba × P. glandulosa*) tissue culture plants were sourced from the National Engineering Research Center of Tree Breeding and Ecological Restoration, Beijing Forestry University. Poplar was cultivated in a greenhouse maintained at a 24 ± 1°C temperature, the relative humidity was set at 70%, and the light–dark period was 16 h/8 h. We used 30-day-old tissue-cultured 84K grown in 1/2 MS medium for genetic transformation via *Agrobacterium*-mediated transformation of leaves.

For the drought treatment experiment, 30-day-old tissue culture plants were transplanted in pots (12 × 12 × 10 cm^3^) with equal amounts of autoclaved soil and water. All plants were grown in the greenhouse (24 ± 1°C) with a 6-h/8-h light/dark photoperiod for 45 days at Beijing Forestry University. Following this, a drought assay was initiated by withholding water for the next 10 days (water withheld until soil moisture content reached 30–35%). Drought treatment was then executed by stopping watering of the soil for 6 days, followed by rewatering the plants for 3 days to assess their recovery.

### Expression analysis of miR159a and other genes in poplar

miRNAs were extracted and cDNA synthesis was carried out using a Plant microRNA Kit and the Mir-X miRNA First-Strand Synthesis kit (Takara, Shiga, Japan) was used for miRNA quantification. Total RNA was extracted and reverse transcription was performed using an E.Z.N.A.^®^ Plant RNA Kit (Omega Bio-Tek, CT, USA) and the PrimeScript RT Reagent Kit with gDNA Eraser (Perfect Real Time) (Takara), respectively. RT–qPCR was carried out with Premix Ex Taq II (Tli RnaseH Plus) (Takara). 5.8S rRNA and 18S rRNA were selected as reference genes. The relative expression levels of genes were calculated as described previously [65]. Each RT–qPCR assay was carried out in three biological replicates. Primers are listed in Supplementary Data Table S10.

### Sequence analysis, computational prediction of miR159a targets, and 5′ RACE

We searched the miRBase database (http://www.mirbase.org/search/) using the keyword ‘miR159a’ to retrieve miR159a in poplar. The sequences of miR159a precursor were downloaded and used for designing primers. psRNATarget was used to predict the target genes of miR159a, as previously described [66]. They were functionally annotated in Phytozome.

5′ RACE was conducted using the GeneRacer Kit (Invitrogen Life Technologies, CA, USA) and the cDNA samples were amplified by nested PCR. The 5′ RACE and gene-specific outer primers M2-OR and M2-2OR were used for the first cycle of nested PCR, followed by the second round of nested PCR, using the 5′ RACE and gene-specific inner primers M2-IR and M2-2IR. Amplification products were purified and cloned into TOPO vector (TransGen Biotech, Beijing, China), which were sequenced at RuiBiotech (Beijing, China).

### Gene vector construction and transformation

The NCBI Primer-BLAST website was used to design gene-specific primers to amplify the poplar miR159a precursor sequence, which was ligated to the pCAMBIA2300 vector. miR159a-STTM was constructed as previously described [67]. The constructs were transformed into *Agrobacterium* and leaves of 84K were transformed using the leaf disc method. Transgenic plants were screened in culture medium containing kanamycin (30 mg/l) and validated by PCR and RT–qPCR as mentioned above.

### Determination of abscisic acid content

The MetWare assay on the AB Sciex QTRAP 6500 LC–MS/MS platform was used to determine ABA content (D6 abscisic acid as an internal standard), as described previously [68].

### Drought stress treatment

We selected 45-day-old transplanted OX159a lines (OX159a-6, OX159a-12, and OX159a-20), miR159a-STTM lines (STTM-3, STTM-7, and STTM-10), and WT lines for drought treatment. The drought assay was performed as described above. We observed and recorded the plants’ growth status in the small pots every 2 days. Soil water content was measured using a Moisture Meter type HH2.

The drought experiment was performed using WT, OX159a, and miR159a-STTM line leaves *in vitro*. A total of 21 plants with the same growth status were selected for the experiment, where three plants per line were used as biological replicates. Leaves in the same position of each plant were measured three times. Mature leaves of each poplar were sampled for measurement of relative water content loss rate at different time points, including 1, 2, 3, 6, 12, 24, and 30 h, as described previously [69]. There were at least three replicates for each treatment.

### Relative electrical conductivity and chlorophyll measurements

Leaves were sampled from the same morphological position of the control and drought-treated OX159a and WT lines and then made into 1-cm^2^ small discs. Each of the leaves was represented by four small discs and the REC of the supernatant was determined using a DDS-307 (Leici-DDS-307A, Shanghai, China) and then calculated according to the formula: relative ion leakage = C1/C2 × 100% (C1 represents the pre-boiling water bath conductivity and C2 represents the post-boiling water bath conductivity). A portable chlorophyll meter (SPAD-502Plus, Konica Minolta, Japan) was used to measure the relative chlorophyll content of the OX159a and WT lines in the fifth to seventh leaves.

### Determination of several ROS physiological indicators and DAB and trypan blue staining

The integrity of the cell membrane and cell survival were examined by trypan blue staining. Plant leaves were immersed in a trypan blue solution after dehydration for 6 days and were then placed in 70% ethanol solution and photographed.

To detect the production of H_2_O_2_, WT and OX159aleaves were immersed in DAB solution after being dehydrated for 6 days [70], and then placed in 70% ethanol solution and photographed.

POD, SOD, and MDA enzyme activities were measured using the Solarbao kit (Beijing, China). POD enzyme activity was calculated according to the formula POD (U/g fresh weight) = 7133 × △A/W (ΔA=A2-A1, A1 represents the absorbance value at the 470 nm wavelength for 30 s, and A2 represents the absorbance value at the 470 nm wavelength after 30 s for 1 min. W represents sample quality), SOD enzyme activity was calculated using the formula 11.11 × IP / (1 − IP) / W × F (IP represents percentage inhibition; F represents sample dilution factor; W represents sample quality), and MDA was calculated using the formula 32.258 × (A532 − A600)/W (W represents sample quality).

### Leaf gas exchange measurements in OX159a lines and wild type in greenhouse

The net photosynthetic rate (*A*), transpiration rate (*E*) and stomatal conductance (Gsw) from the top to the fifth to seventh healthy leaves in OX159a lines and WT were measured at 9 to 11 a.m. An Li-6800 portable photosynthesizer was set to the leaf chamber of the natural light source and there were six replicates for each treatment. The ratio *A*/*E* was used to calculate WUE. Parameter settings were as follows: CO_2_ concentration = 400 μmol/mol; flow = 1400 μmol/s.

### Stomatal parameter measurement

Stomatal pores of OX159a, miR159a-STTM, and WT lines were photographed with an inverted biologic microscope (DMi8, Leica, Wetzlar, Germany). The nail polish imprinting method was used to determine the characteristic parameters of stomatal morphology. The underside of the leaves was wiped smooth and colorless nail varnish was then applied to polish the leaves evenly. The dried nail varnish was peeled off for observation under 20×, 40×, and 63× fields of view of the microscope. Finally, the number of stomata was counted and their size was measured under the 20× and 40× fields of view of the microscope, respectively. Stomatal number/field of view area was used as a statistical measure of stomatal density (stomatal density, SD).

### Histological analyses

Poplar stem (3-month-old poplars in pots) internodes were cut into 2-mm sections, and placed in ethylene glycol ethyl ether acetate I (37°C, 6 h) and ethylene ether acetate II overnight (37°C). Then, the internodes were placed in ethylene glycol ethyl ether acetate III (10–15 min at room temperature) and ethylene glycol ethyl ether acetate IV (10–15 min at room temperature). After this, the internodes were rinsed with running water. Finally, the sections were placed in toluidine blue staining solution (~2 min), and washed and observed under an inverted microscope (Nikon Eclipse E100). The sections were placed in an oven (60°C) for drying after washing with running water. Finally, neutral gum was used for sealing the sections. A microscope (Nikon DS-U3) was used to observe, collect, and analyze the images.

### Transcriptomic analysis

We performed transcriptome analysis using leaves of WT and OX159a plants grown in soil for 45 days (under adequate water conditions and subjected to 6 days of drought), with three biological replicates. Trizol (Invitrogen Life Technologies, CA, USA) was used to extract RNA. A total of 12 cDNA libraries were constructed and sequenced on the DNBSEQ platform at Beijing Genomics Institute, as described previously [71]. High-quality reads were aligned to the 84K genome [72] by HISAT [73] and Bowtie 2 with default parameters [74]. Then, the gene expression level of each sample was calculated by RSEM. The FPKM was obtained based on the length and mapped read number of identified genes. DEGs were identified with an adjusted *P*-value <.05 and absolute log_2_ fold change ≥1. DEG function was annotated based on Phytozome JGI and the KEGG and GO databases with default parameters.

### Statistical analysis

The data were analyzed using GraphPad Prism 9 software. The results show the mean of three biological replicates.

## Supplementary Material

Web_Material_uhad221

## References

[1] Zhang H , Zhu J, Gong Z. et al. Abiotic stress responses in plants. Nat Rev Genet. 2022;23:104–19 34561623 10.1038/s41576-021-00413-0

[2] Choat B , Jansen S, Brodribb TJ. et al. Global convergence in the vulnerability of forests to drought. Nature. 2012;491:752–5 23172141 10.1038/nature11688

[3] Watters TR , Robinson MS, Beyer RA. et al. Evidence of recent thrust faulting on the moon revealed by the Lunar Reconnaissance Orbiter Camera. Science. 2010;329:936–40 20724632 10.1126/science.1189590

[4] Fichot R , Brignolas F, Cochard H. et al. Vulnerability to drought-induced cavitation in poplars: synthesis and future opportunities. Plant Cell Environ. 2015;38:1233–51 25444560 10.1111/pce.12491

[5] Fang X , Zhao G, Zhang S. et al. Chloroplast-to-nucleus signaling regulate microRNA biogenesis in *Arabidopsis*. Dev Cell. 2019;48:371–382.e4 30595534 10.1016/j.devcel.2018.11.046

[6] Chen MSAX , Murata N. Enhancement of tolerance of abiotic stress by metabolic engineering of betaines and other compatible solutes. Curr Opin Plant Biol. 2002;5:250–7 11960744 10.1016/s1369-5266(02)00255-8

[7] Lin Z , Wang YL, Cheng LS. et al. Mutual regulation of ROS accumulation and cell autophagy in wheat roots under hypoxia stress. Plant Physiol Biochem. 2021;158:91–102 33302125 10.1016/j.plaphy.2020.11.049

[8] Su Y , Li HG, Wang Y. et al. Poplar miR472a targeting NBS-LRRs is involved in effective defence against the necrotrophic fungus *Cytospora chrysosperma*. J Exp Bot. 2018;69:5519–30 30124931 10.1093/jxb/ery304

[9] Xin M , Wang Y, Yao Y. et al. Diverse set of microRNAs are responsive to powdery mildew infection and heat stress in wheat (*Triticum aestivum* L.). BMC Plant Biol. 2010;10:123–3 20573268 10.1186/1471-2229-10-123 PMC3095282

[10] Li L , Yi H, Xue M. et al. MiR398 and miR395 are involved in response to SO_2_ stress in *Arabidopsis thaliana*. Ecotoxicology. 2017;26:1181–7 28819808 10.1007/s10646-017-1843-y

[11] Zhao J , Yuan S, Zhou M. et al. Transgenic creeping bentgrass overexpressing Osa-miR393a exhibits altered plant development and improved multiple stress tolerance. Plant Biotechnol J. 2019;17:233–51 29873883 10.1111/pbi.12960 PMC6330543

[12] Jiao Z , Lian C, Han S. et al. PtmiR169o plays a positive role in regulating drought tolerance and growth by targeting the PtNF-YA6 gene in poplar. Environ Exp Bot. 2021;189:104549

[13] Liu Q , Wang Z, Yu S. et al. Pu-miR172d regulates stomatal density and water-use efficiency via targeting PuGTL1 in poplar. J Exp Bot. 2021;72:1370–83 33098429 10.1093/jxb/eraa493

[14] Wu L , Liu D, Wu J. et al. Regulation of FLOWERING LOCUS T by a microRNA in *Brachypodium distachyon*. Plant Cell. 2013;25:4363–77 24285787 10.1105/tpc.113.118620 PMC3875723

[15] Kutter C , Schob H, Stadler M. et al. MicroRNA-mediated regulation of stomatal development in *Arabidopsis*. Plant Cell. 2007;19:2417–29 17704216 10.1105/tpc.107.050377 PMC2002609

[16] Millar AA , Lohe A, Wong G. Biology and function of miR159 in plants. Plants (Basel). 2019;8:255 31366066 10.3390/plants8080255 PMC6724108

[17] Akdogan G , Tufekci ED, Uranbey S. et al. miRNA-based drought regulation in wheat. Funct Integr Genomics. 2016;16:221–33 26141043 10.1007/s10142-015-0452-1

[18] López-Galiano MJ , García-Robles I, González-Hernández AI. et al. Expression of miR159 is altered in tomato plants undergoing drought stress. Plants (Basel). 2019;8:201 31269704 10.3390/plants8070201 PMC6681330

[19] Zhang B . MicroRNA: a new target for improving plant tolerance to abiotic stress. J Exp Bot. 2015;66:1749–61 25697792 10.1093/jxb/erv013 PMC4669559

[20] Narusaka M , Ohtani M, Demura T. et al. Development of a model system comprising *Populus* as a model tree and *Colletotrichum gloeosporioides* as a model pathogen for studying host-pathogen interactions. Plant Biotechnol. 2012;29:511–4

[21] Fang Y , Wang D, Xiao L. et al. Allelic variation in transcription factor PtoWRKY68 contributes to drought tolerance in *Populus*. Plant Physiol. 2023;193:736–55 37247391 10.1093/plphys/kiad315 PMC10469405

[22] Fang Q , Wang X, Wang H. et al. The poplar R2R3 MYB transcription factor PtrMYB94 coordinates with abscisic acid signaling to improve drought tolerance in plants. Tree Physiol. 2020;40:46–59 31728530 10.1093/treephys/tpz113

[23] Wang S , Fan Y, Du S. et al. PtaERF194 inhibits plant growth and enhances drought tolerance in poplar. Tree Physiol. 2022;42:1678–92 35220440 10.1093/treephys/tpac026

[24] Ahmed W , Xia Y, Zhang H. et al. Identification of conserved and novel miRNAs responsive to heat stress in flowering Chinese cabbage using high-throughput sequencing. Sci Rep. 2019;9:14922 31624298 10.1038/s41598-019-51443-y PMC6797766

[25] Castillo-Argaez R , Schaffer B, Vazquez A. et al. Leaf gas exchange and stable carbon isotope composition of redbay and avocado trees in response to laurel wilt or drought stress. Environ Exp Bot. 2020;171:103948

[26] Tcherkez G , Limami AM. Net photosynthetic CO_2_ assimilation: more than just CO_2_ and O_2_ reduction cycles. New Phytol. 2019;223:520–9 30927445 10.1111/nph.15828

[27] Li S , Lin YCJ, Wang P. et al. The AREB1 transcription factor influences histone acetylation to regulate drought responses and tolerance in *Populus trichocarpa*. Plant Cell. 2019;31:663–86 30538157 10.1105/tpc.18.00437 PMC6482633

[28] Chico JM , Lechner E, Fernandez-Barbero G. et al. CUL3^BPM^ E3 ubiquitin ligases regulate MYC2, MYC3, and MYC4 stability and JA responses. Proc Natl Acad Sci USA. 2020;117:6205–15 32123086 10.1073/pnas.1912199117 PMC7084108

[29] Wang TJ , Huang S, Zhang A. et al. JMJ17-WRKY40 and HY5-ABI5 modules regulate the expression of ABA-responsive genes in *Arabidopsis*. New Phytol. 2021;230:567–84 33423315 10.1111/nph.17177

[30] Li XW , Zhu YL, Chen CY. et al. Cloning and characterization of two chlorophyll A/B binding protein genes and analysis of their gene family in *Camellia sinensis*. Sci Rep. 2020;10:4602 32165676 10.1038/s41598-020-61317-3 PMC7067855

[31] Khanna R , Kronmiller B, Maszle DR. et al. The *Arabidopsis* B-box zinc finger family. Plant Cell. 2009;21:3416–20 19920209 10.1105/tpc.109.069088 PMC2798317

[32] Nagaoka S , Takano T. Salt tolerance-related protein STO binds to a Myb transcription factor homologue and confers salt tolerance in *Arabidopsis*. J Exp Bot. 2003;54:2231–7 12909688 10.1093/jxb/erg241

[33] Addo-Quaye C , Eshoo TW, Bartel DP. et al. Endogenous siRNA and miRNA targets identified by sequencing of the *Arabidopsis* degradome. Curr Biol. 2008;18:758–62 18472421 10.1016/j.cub.2008.04.042 PMC2583427

[34] Song QX , Liu YF, Hu XY. et al. Identification of miRNAs and their target genes in developing soybean seeds by deep sequencing. BMC Plant Biol. 2011;11:5 21219599 10.1186/1471-2229-11-5 PMC3023735

[35] Liu N , Tu L, Tang W. et al. Small RNA and degradome profiling reveals a role for miRNAs and their targets in the developing fibers of *Gossypium barbadense*. Plant J. 2014;80:331–44 25131375 10.1111/tpj.12636

[36] Zhang J , Zeng R, Chen J. et al. Identification of conserved microRNAs and their targets from *Solanum lycopersicum* Mill. Gene. 2008;423:1–7 18602455 10.1016/j.gene.2008.05.023

[37] An FM , Chan MT. Transcriptome-wide characterization of miRNA-directed and non-miRNA-directed endonucleolytic cleavage using degradome analysis under low ambient temperature in *Phalaenopsis aphrodite* subsp. *formosana*. Plant Cell Physiol. 2012;53:1737–50 22904110 10.1093/pcp/pcs118

[38] Luo X , Gao Z, Shi T. et al. Identification of miRNAs and their target genes in peach (*Prunus persica* L.) using high-throughput sequencing and degradome analysis. PLoS One. 2013;8:e79090 24236092 10.1371/journal.pone.0079090 PMC3827290

[39] Sun F , Guo G, Du J. et al. Whole-genome discovery of miRNAs and their targets in wheat (*Triticum aestivum* L.). BMC Plant Biol. 2014;14:142 24885911 10.1186/1471-2229-14-142 PMC4048363

[40] Li YF , Zheng Y, Addo-Quaye C. et al. Transcriptome-wide identification of microRNA targets in rice. Plant J. 2010;62:742–59 20202174 10.1111/j.1365-313X.2010.04187.x

[41] Curaba J , Spriggs A, Taylor J. et al. miRNA regulation in the early development of barley seed. BMC Plant Biol. 2012;12:120 22838835 10.1186/1471-2229-12-120 PMC3443071

[42] Park S , Grabau E. Bypassing miRNA-mediated gene regulation under drought stress: alternative splicing affects CSD1 gene expression. Plant Mol Biol. 2017;95:243–52 28776286 10.1007/s11103-017-0642-4

[43] Shen X , He J, Ping Y. et al. The positive feedback regulatory loop of miR160-auxin response factor 17-HYPONASTIC LEAVES 1 mediates drought tolerance in apple trees. Plant Physiol. 2022;188:1686–708 34893896 10.1093/plphys/kiab565 PMC8896624

[44] Konings AG , Saatchi SS, Frankenberg C. et al. Detecting forest response to droughts with global observations of vegetation water content. Glob Chang Biol. 2021;27:6005–24 34478589 10.1111/gcb.15872 PMC9293345

[45] Liu H , Tian X, Li Y. et al. Microarray-based analysis of stress-regulated microRNAs in *Arabidopsis thaliana*. RNA. 2008;14:836–43 18356539 10.1261/rna.895308 PMC2327369

[46] Wang Y , Sun F, Cao H. et al. TamiR159 directed wheat TaGAMYB cleavage and its involvement in anther development and heat response. PLoS One. 2012;7:e48445 23133634 10.1371/journal.pone.0048445 PMC3486836

[47] Pieczynski M , Marczewski W, Hennig J. et al. Down-regulation of *CBP80* gene expression as a strategy to engineer a drought-tolerant potato. Plant Biotechnol J. 2013;11:459–69 23231480 10.1111/pbi.12032

[48] Grondin A , Rodrigues O, Verdoucq L. et al. Aquaporins contribute to ABA-triggered stomatal closure through OST1-mediated phosphorylation. Plant Cell. 2015;27:1945–54 26163575 10.1105/tpc.15.00421 PMC4531361

[49] Jalakas P , Merilo E, Kollist H. et al. ABA-mediated regulation of stomatal density is OST1-independent. Plant Direct. 2018;2:e00082 31245747 10.1002/pld3.82 PMC6508810

[50] Mustilli A , Merlot S, Vavasseur A. et al. *Arabidopsis* OST1 protein kinase mediates the regulation of stomatal aperture by abscisic acid and acts upstream of reactive oxygen species production. Plant Cell. 2002;14:3089–99 12468729 10.1105/tpc.007906 PMC151204

[51] Yin Y , Adachi Y, Nakamura Y. et al. Involvement of OST1 protein kinase and PYR/PYL/RCAR receptors in methyl jasmonate-induced stomatal closure in *Arabidopsis* guard cells. Plant Cell Physiol. 2016;57:1779–90 27354421 10.1093/pcp/pcw102

[52] Yoshida T , Christmann A, Yamaguchi-Shinozaki K. et al. Revisiting the basal role of ABA-roles outside of stress. Trends Plant Sci. 2019;24:625–35 31153771 10.1016/j.tplants.2019.04.008

[53] Fisher JB , Goldstein G, Jones TJ. et al. Wood vessel diameter is related to elevation and genotype in the Hawaiian tree *Metrosideros polymorpha* (Myrtaceae). Am J Bot. 2007;94:709–15 21636440 10.3732/ajb.94.5.709

[54] Huang S , Van Aken O, Schwarzländer M. et al. The roles of mitochondrial reactive oxygen species in cellular signaling and stress response in plants. Plant Physiol. 2016;171:1551–9 27021189 10.1104/pp.16.00166 PMC4936549

[55] Qi J , Song CP, Wang B. et al. Reactive oxygen species signaling and stomatal movement in plant responses to drought stress and pathogen attack. J Integr Plant Biol. 2018;60:805–26 29660240 10.1111/jipb.12654

[56] Noctor G , Mhamdi A, Foyer CH. The roles of reactive oxygen metabolism in drought: not so cut and dried. Plant Physiol. 2014;164:1636–48 24715539 10.1104/pp.113.233478 PMC3982730

[57] Suzuki N , Koussevitzky S, Mittler R. et al. ROS and redox signaling in the response of plants to abiotic stress. Plant Cell Environ. 2012;35:259–70 21486305 10.1111/j.1365-3040.2011.02336.x

[58] Wang K , Bu T, Cheng Q. et al. Two homologous LHY pairs negatively control soybean drought tolerance by repressing the abscisic acid responses. New Phytol. 2021;229:2660–75 33095906 10.1111/nph.17019

[59] Shuai P , Liang D, Zhang Z. et al. Identification of drought-responsive and novel *Populus trichocarpa* microRNAs by high-throughput sequencing and their targets using degradome analysis. BMC Genomics. 2013;14:233 23570526 10.1186/1471-2164-14-233 PMC3630063

[60] Tsuji H , Aya K, Ueguchi-Tanaka M. et al. GAMYB controls different sets of genes and is differentially regulated by microRNA in aleurone cells and anthers. Plant J. 2006;47:427–44 16792694 10.1111/j.1365-313X.2006.02795.x

[61] Cuperus JT , Fahlgren N, Carrington JC. Evolution and functional diversification of *MIRNA* genes. Plant Cell. 2011;23:431–42 21317375 10.1105/tpc.110.082784 PMC3077775

[62] Basso MF , Ferreira PCG, Kobayashi AK. et al. MicroRNAs and new biotechnological tools for its modulation and improving stress tolerance in plants. Plant Biotechnol J. 2019;17:1482–500 30947398 10.1111/pbi.13116 PMC6662102

[63] Zhou J , Zhang R, Jia X. et al. CRISPR-Cas9 mediated OsMIR168a knockout reveals its pleiotropy in rice. Plant Biotechnol J. 2022;20:310–22 34555252 10.1111/pbi.13713 PMC8753357

[64] Cheah BH , Nadarajah K, Divate MD. et al. Identification of four functionally important microRNA families with contrasting differential expression profiles between drought-tolerant and susceptible rice leaf at vegetative stage. BMC Genomics. 2015;16:692 26369665 10.1186/s12864-015-1851-3 PMC4570225

[65] Livak KJ , Schmittgen TD. Analysis of relative gene expression data using real-time quantitative PCR and the 2^-ΔΔCT^ method. Methods. 2001;25:402–8 11846609 10.1006/meth.2001.1262

[66] Dai X , Zhuang Z, Zhao PX. psRNATarget: a plant small RNA target analysis server (2017 release). Nucleic Acids Res. 2018;46:W49–54 29718424 10.1093/nar/gky316 PMC6030838

[67] Tang G , Yan J, Gu Y. et al. Construction of short tandem target mimic (STTM) to block the functions of plant and animal microRNAs. Methods. 2012;58:118–25 23098881 10.1016/j.ymeth.2012.10.006 PMC3631596

[68] Rao S , Tian Y, Zhang C. et al. The JASMONATE ZIM-domain-OPEN STOMATA1 cascade integrates jasmonic acid and abscisic acid signaling to regulate drought tolerance by mediating stomatal closure in poplar. J Exp Bot. 2023;74:443–57 36260345 10.1093/jxb/erac418

[69] He F , Wang HL, Li HG. et al. PeCHYR1, a ubiquitin E3 ligase from *Populus euphratica*, enhances drought tolerance via ABA-induced stomatal closure by ROS production in *Populus*. Plant Biotechnol J. 2018;16:1514–28 29406575 10.1111/pbi.12893 PMC6041450

[70] Ding S , Zhang B, Qin F. *Arabidopsis* RZFP34/CHYR1, a ubiquitin E3 ligase, regulates stomatal movement and drought tolerance via SnRK2.6-mediated phosphorylation. Plant Cell. 2015;27:3228–44 26508764 10.1105/tpc.15.00321 PMC4682294

[71] Drmanac R , Sparks AB, Callow MJ. et al. Human genome sequencing using unchained base reads on self-assembling DNA nanoarrays. Science. 2010;327:78–81 19892942 10.1126/science.1181498

[72] Qiu D , Bai S, Ma J. et al. The genome of *Populus alba* × *Populus tremula* var. *glandulosa* clone 84K. DNA Res. 2019;26:423–31 31580414 10.1093/dnares/dsz020 PMC6796506

[73] Kim D , Langmead B, Salzberg SL. HISAT: a fast spliced aligner with low memory requirements. Nat Methods. 2015;12:357–60 25751142 10.1038/nmeth.3317 PMC4655817

[74] Langmead B , Salzberg SL. Fast gapped-read alignment with Bowtie 2. Nat Methods. 2012;9:357–9 22388286 10.1038/nmeth.1923 PMC3322381

