# Correction to: Function identification of miR159a, a positive regulator during poplar resistance to drought stress

**DOI:** 10.1093/hr/uhag385

**Published:** 2026-10-05

This is a correction to: Tiantian Fu, Chun Wang, Yuzhang Yang, Xiaoqian Yang, Jing Wang, Lichun Zhang, Zeqi Wang, Yanwei Wang, Function identification of miR159a, a positive regulator during poplar resistance to drought stress, *Horticulture Research*, Volume 10, Issue 12, December 2023, uhad221, https://doi.org/10.1093/hr/uhad221

In the originally published version of this manuscript, the image from the OX159a-20 line in Figure 5c was mistakenly used for both the OX159a-12 and OX159a-20 lines, due to an error that occurred during figure assembly.

The authors apologize for this oversight and would like to emphasize that the correction does not affect the analysis or the conclusions of the study.

The corrected image of OX159a-12 is provided below:



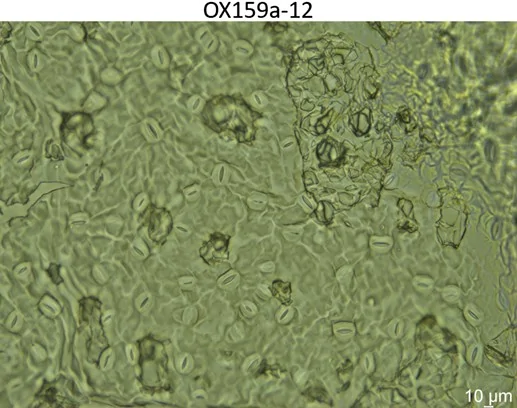



This error has been corrected only in this correction notice to preserve the version of record.

