# Supplementary material for: Function identification of miR159a, a positive regulator during poplar resistance to drought stress
Source: Hortic Res. 2023 Nov 7;10(12):uhad221. doi: 10.1093/hr/uhad221 (PMC10709547; doi:10.1093/hr/uhad221)
Supplement: Web_Material_uhad221 [file web_material_uhad221.zip › Figure_S1-S6.docx]

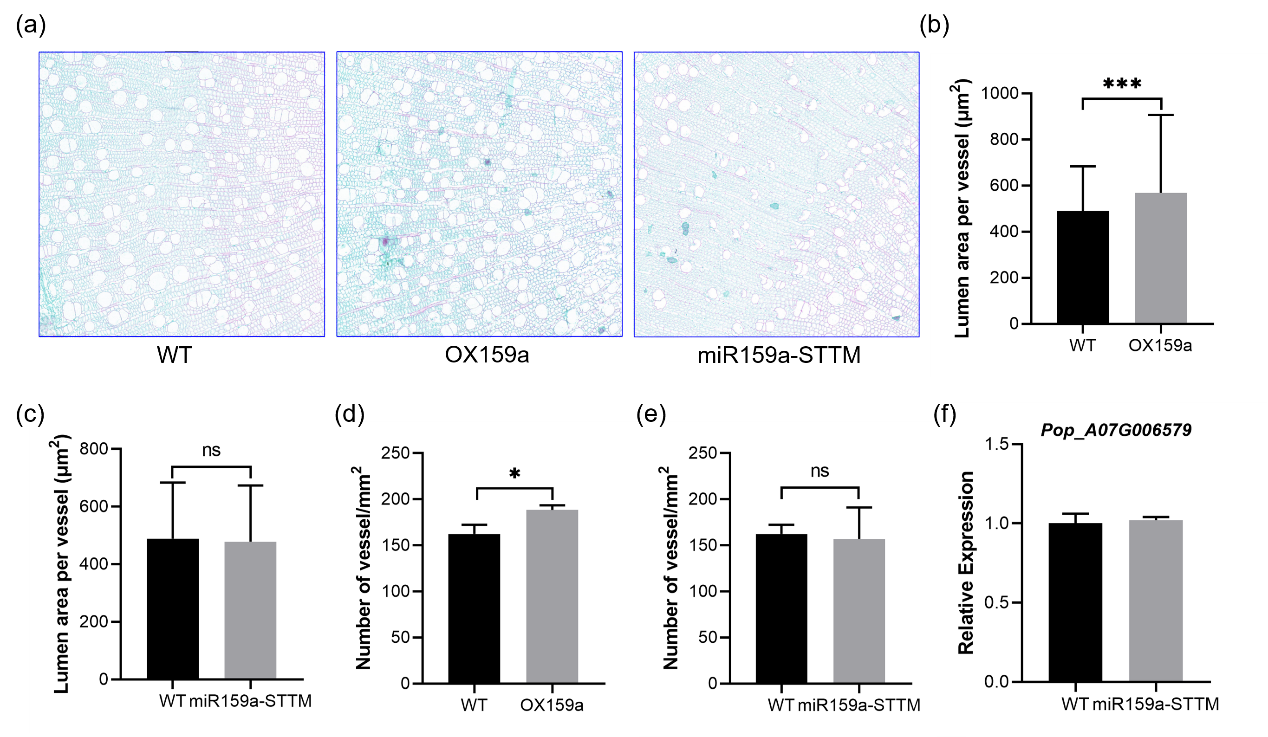


**Figure S1** Overexpressing miR159a affects the Size and number of vessels in xylem tissue of 84k. (a) Stem cross sections of wild-type (WT) and OX159a lines with the last internode. The blue box line in (a) is 1 mm. (b-c) Statistical analysis of mean lumen area of individual vessels (mm^2^). (d-e) Statistical analysis of number of vessel (mm^2^). (f) The expression level of *Pop_A07G006579* (*NAC007*) between miR159a-STTM and WT. (b) number of vessels per cross-sectional area (mm^2^) (c) and area of vessels (mm^2^) per cross-sectional area (mm^2^) (d) using vessel cells from (a). Error bars represent 1 SE of three independent replicates with at least 380 vessel cells for each genotype in each replicate. 18S rRNA was used as the internal reference gene. Error bars: ± SD with three biological replicates. The asterisk symbol represents significant differences (Student *t* test; **P* < 0.05; ***P* < 0.01; ****P* < 0.001).


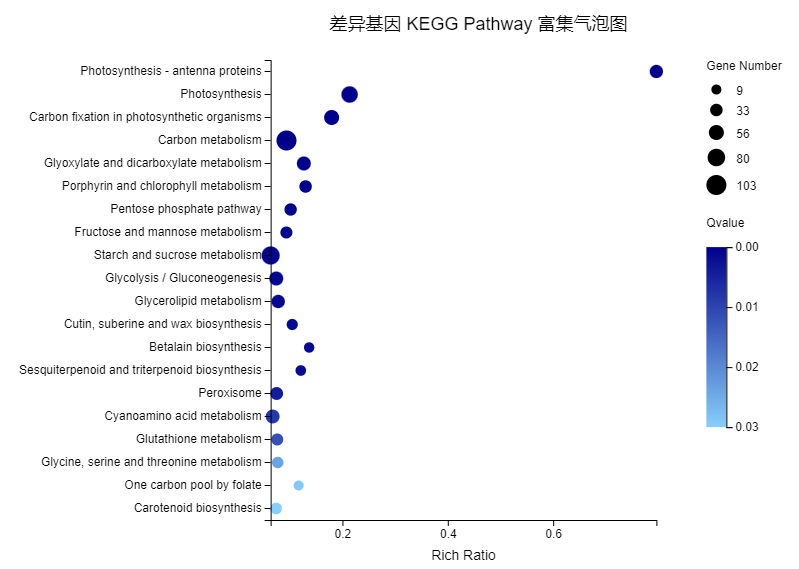


**Figure S2** KEGG pathway enrichment of DEGs in OX159a and WT lines.


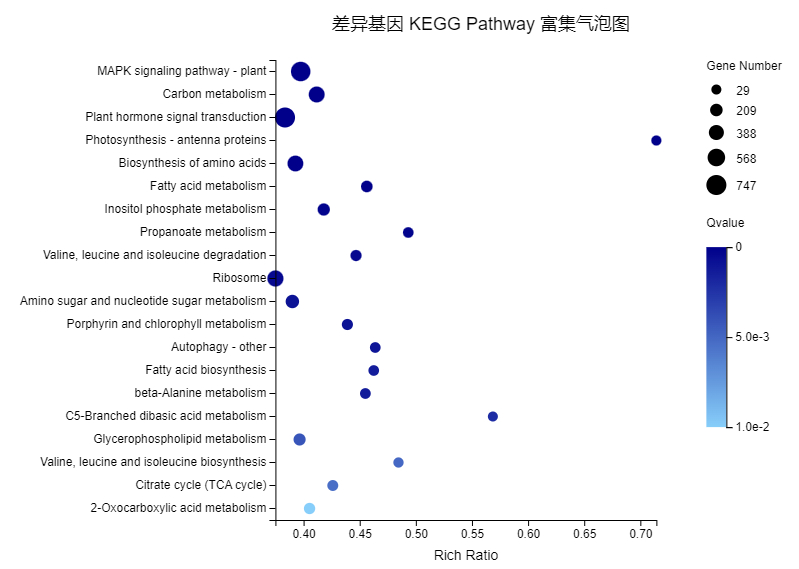


**Figure S3** KEGG pathway enrichment of DEGs in OX159a-12 after 6 d drought treatment.


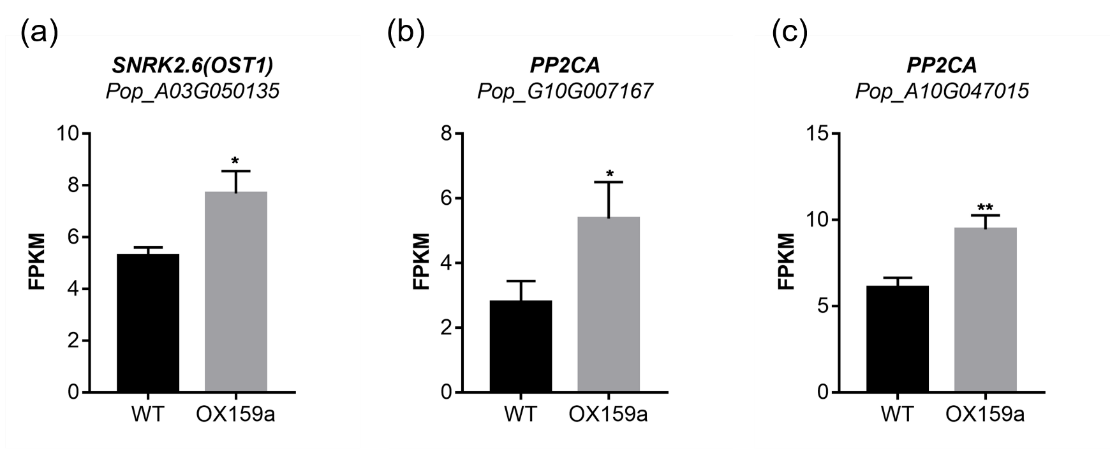


**Figure S4** MiR159a promotes gene expression in the ABA signaling pathway. OX159a-12 line was performed for the analysis considering the most highly expression of miR159a. Error bars indicate ± SD for three biological replicates. The asterisk symbol represents significant differences (Student *t* test; **P* < 0.05; ***P* < 0.01; ****P* < 0.001).


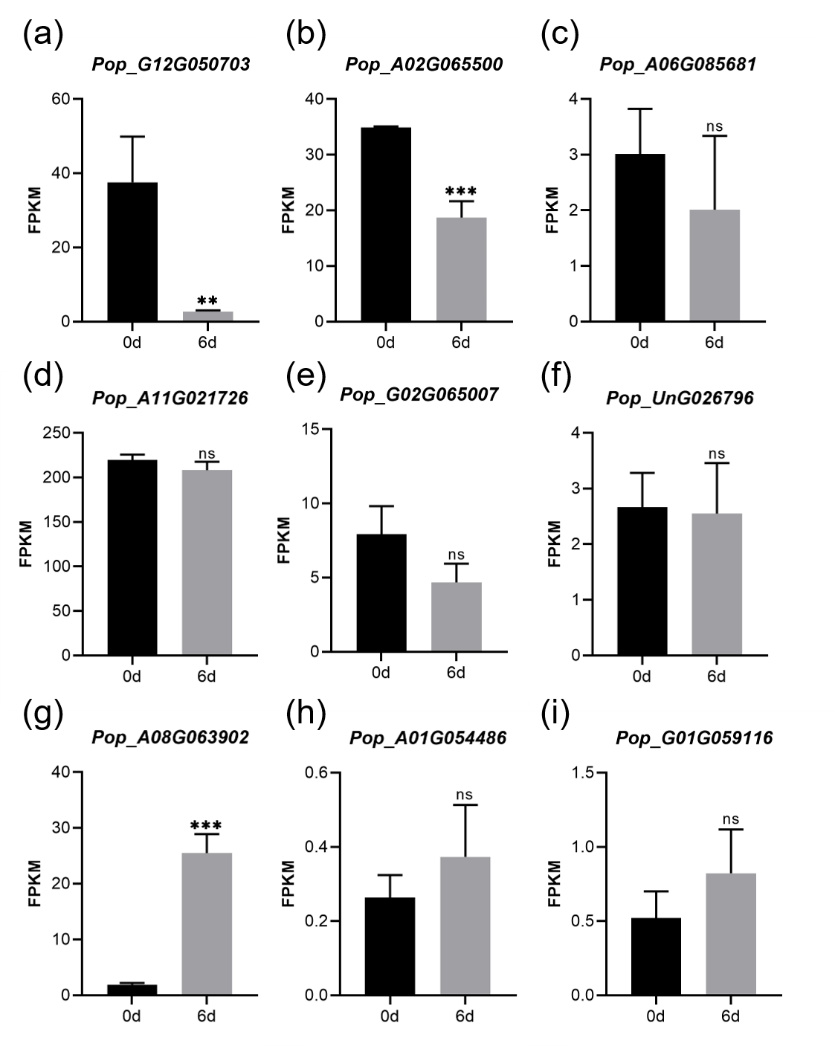


**Figure S5** Expression analysis of other target genes detected by transcriptomics and degradomics under drought stress. Under normal growth conditions (0 d), after 6 d drought treatment (6 d). Error bars indicate ± SD for three biological replicates. The asterisk symbol represents significant differences (Student *t* test; **P* < 0.05; ***P* < 0.01; ****P* < 0.001; no significance (ns)).


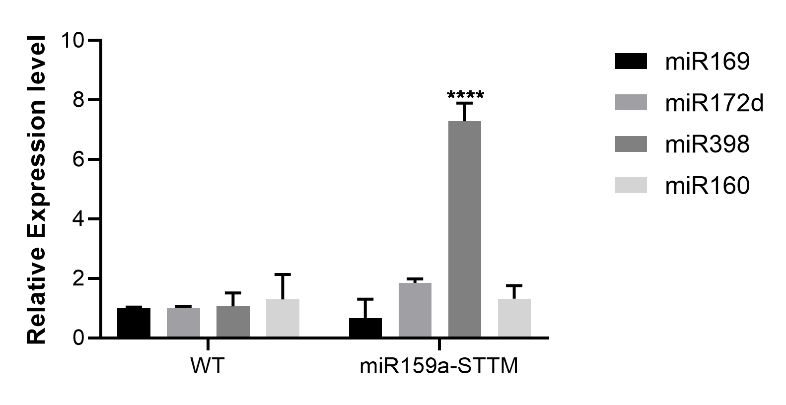


**Figure S6** MiR159a affects the biogenesis of drought-responsive miR172d, miR169o, miR160 and miR398 in poplar. miR159a-STTM line was performed for the analysis considering. Error bars: ± SD with three biological replicates. The asterisk symbol represents significant differences (Student *t* test; **P* < 0.05; ***P* < 0.01; ****P* < 0.001).
